# Is personality reflected in the gestures of second language speakers?

**DOI:** 10.3389/fpsyg.2024.1463063

**Published:** 2024-09-11

**Authors:** Renia Lopez-Ozieblo

**Affiliations:** The Hong Kong Polytechnic University, Kowloon, Hong Kong SAR, China

**Keywords:** personality, gestures, functional classification, proficiency, foreign language learners, anxiety

## Abstract

Previous studies on gestures and personality suggest an ambiguous picture of the effects of the various personality dimensions on the different types of speech-gestures and adaptors. In foreign language learning an additional variable to take into account is proficiency, which some studies have shown to affect gestures. In this study, we explore how various intrinsic variables, including personality, proficiency, gender and age affect the gestures of 61 Cantonese speakers of English as a second language. Participants were asked to narrate a video cartoon. Their proficiency and the frequency of gestures produced was based on those narrations. A functional categorization of gestures was followed, dividing them into semantic and discursive, and that also noted adaptors and micro-gestures, referred to as “flutters.” Personality was self-reported using the 44 question Big-Five inventory. Correlations and a series of generalized linear models were developed to explore the interplay between variables. Agreeableness was found to be positively correlated with semantic gestures; and neuroticism and age were negatively correlated with flutter duration. Contrary to the findings from previous studies, no significant relationships were found between neuroticism and adaptors or semantic gestures, nor between extraversion or openness and semantic gestures. Proficiency and gender had little effect on gestures. As personality has been shown to be an important factor in gesture production in mother tongue speakers, we expected to also see a similar result with foreign language speakers, this not being so suggests that other variables, aside from those tested, should be considered. In particular, the results suggest that emotion and emotional constructs, such as anxiety, self-confidence or empathy, might have a greater impact on gesture production than proficiency or personality, a point which should be taken into account especially in language evaluation contexts or professional contexts with second language speakers.

## 1 Introduction

In the second language (L2) acquisition context, individual-related factors have been extensively studied to understand how they impact acquisition. Some of these factors, including gender (Dong et al., 2023), are related to personality (Dewaele, 2012). These factors influence motivation, affect and emotion which in turn impact the learner’s attitude toward the new language, the learning process and acquisition (Dörnyei, 2005). Personality and how individuals gesture are thought to be related (Hostetter and Potthoff, 2012) as gestures are idiosyncratic (McNeill, 1992). In the second language context, gestures are relevant as they are cognitive and communicative tools (Gullberg, 2022), but it is not known whether proficiency or personality weigh more in how gesture is produced, thus understanding these relationships could help educators interpret learners’ needs and guide them to use these communicative resources optimally.

In this study, we explore the relationships between proficiency, personality and gestures in 61 Cantonese speakers of English as a second language, we also analyze age and gender as two potential variables in gesture production. Age was not one of our original variables but was added after the data was analyzed, in response to comments from our participants.

### 1.1 Personality

Individual differences in human behavior are attributed to personality as well as to other factors such as age, gender or social experiences (Lubinski, 2000; Özer and Göksun, 2020). Personality is reflected in a series of traits that have been grouped in multi-trait models to provide personality profiles reported to be universal. Most models are based on five dimensions, the Big Five (Goldberg, 1992) that include: openness to new experiences (e.g., open to new ideas and experiences), extraversion (e.g., outgoing, adventurous), agreeableness (e.g., trust, altruism, compliance), conscientiousness (e.g., competence, self-discipline) and neuroticism (e.g., anxiety, depression, self-consciousness; Costa and McCrae, 1992a; John and Srivastava, 1999). The following facets and correlated trait adjectives are associated to each of these five dimensions:

- Individuals high in neuroticism often exhibit anxiety, becoming tense in situations that others may find non-threatening. Self-consciousness and low self-esteem are also common, with these individuals being shy and uncomfortable in social situations. Impulsiveness, characterized by moodiness and unpredictable behavior, is another facet of neuroticism.
- Individuals high in extraversion are often warm and outgoing, enjoying social interactions and being around others. Assertiveness is another key trait, with these individuals being forceful and confident in expressing their opinions. They are also typically active and energetic, seeking out excitement and adventure.
- Openness to experience is characterized by curiosity, creativity, and a preference for novelty and variety. Individuals high in openness often have a rich fantasy life and are imaginative. They also appreciate esthetics and are often artistic.
- Individuals high in agreeableness are often kind, trusting and forgiving, believing in the goodness of others. They are also straightforward, altruistic, honest and not demanding. These individuals are also compliant, being cooperative and not stubborn.
- Conscientiousness is characterized by organization, responsibility, and dependability. Individuals high in conscientiousness are often competent and efficient, being capable and reliable in their work. They also value order, being organized, methodical and deliberative, thinking carefully before acting.

Over the past few decades, a self-completed questionnaire, the 44 question Big-Five inventory (BFI; John et al., 2008), based on the earlier Five Factor Model (Costa and McCrae, 1992a), has become the most widely used instrument to measure personality. Other personality tests include the NEO-60 (Costa and McCrae, 1985) and NEO-PI-R (Costa and McCrae, 1992b) which also measure the Big Five traits. The Eysenck Personality Questionnaire (EPI; Eysenck, 1975) measures different traits, although extraversion and neuroticism are covered. Other models include the Myers Briggs Type indicator (Myers-Briggs Type Indicator®, n.d.) as well as six- and seven-dimension models (Zhou et al., 2009). The BFI is most used today and is standard in psychology studies (John, 2021; Neff et al., 2011).

The 44 BFI has been administered to over 15,000 people in 55 countries and cultures to obtain a global picture of personality profiles by gender (Schmitt et al., 2008). The study concluded that personality profiles were culture-dependent, modulated by socioeconomic factors, education and wealth. These factors were stronger personality predictors than gender. Globally, women scored higher than men in neuroticism, agreeableness, extraversion and conscientiousness while openness results were mixed. Hong Kong, the location of this study, was included as one of those 55 cultures, noting slightly higher levels of extraversion in men than women (contrary to the global average). If gender differences affect personality these could also be reflected in the gestures observed in men and women.

#### 1.1.1 Personality and L2 speakers

Individuals’ scores on personality dimensions are influenced by the language in which tests were taken (Dylman and Zakrisson, 2023; Veltkamp et al., 2012), suggesting that personality might vary across languages in bi- or multilingual speakers (Li, 2021; Chen and Bond, 2010; Rosselli et al., 2017; Veltkamp et al., 2012). Bilinguals consistently report experiencing a sense of being a different person when speaking their first language compared to their second language (Rosselli et al., 2017), which could be reflected in how they gesture. The shifts in personality observed in bilinguals can be influenced by various factors, including perceived cultural norms associated with each language, language priming effects, and even the ethnicity of the person they are conversing with (Chen and Bond, 2010). These influences contribute to the development of distinct personality patterns, feelings and self-perception as speakers switch from one language to another (Dewaele and Nakano, 2013). In addition, gender related factors, such as social norms and stereotypes can shape personalities (Dong et al., 2023), as can physiological and neurological differences that also impact learning abilities (Ullman, 2001).

In speakers of L2, a number of personality traits are thought to impact L2 communication. MacIntyre and Charos (1996) suggest that extraversion can positively impact L2 communication, as extraverts are more willing to engage in and seek out interactions. On the other hand, anxiety, linked to neuroticism, can hinder L2 communication. However, as Dewaele (2012) pointed out, these results are not always replicated, with situation variables affecting communication more than personality alone. Extraverts score higher in oral fluency measures but not necessarily in tests where fluency is not evaluated (Dewaele and Furnham, 1999). Individuals with higher levels of neuroticism have been found to perform better in oral tests (Robinson et al., 1994) but they also experience more anxiety (Dewaele, 2002). Anxiety affects L2 learners mostly when speaking (MacIntyre and Gardner, 1994), potentially “freezing” their ability to communicate (Dewaele, 2012, p. 50). Higher conscientiousness levels are related to hard-working individuals who are precise users of lexicon and syntax (Ehrman, 2008). Openness has been linked to frequency of L2 use, as these individuals seek more opportunities to practice their L2 language (Dewaele, 2012). Agreeableness has been associated to empathy (Nagels et al., 2015), a complex trait key in language learning (Angelovska et al., 2021) as it helps L2 speakers understand the L2 language by relating to the cultural and emotional context, through verbal and non-verbal cues such as gestures. Therefore, understanding whether and how gestures are affected by personality might provide L2 pedagogues and interlocutors with an additional resource to identify individual L2 speakers’ circumstances.

### 1.2 Gestures

Gestures, for the purposes of this study, are communicative movements of the fingers, hands and arms that occur with speech. Initial categorizations of gesture followed Ekman and Friesen (1969) semiotic and functional five types: emblems, codified gestures not needing speech to be understood (e.g., the thumbs up sign); illustrators, movements usually co-occurring with speech, used to describe or add emphasis (e.g., holding an imaginary fish between one’s hands to indicate how big it was or waging a finger to indicate disagreement); regulators, gestures that control the flow of the communication (e.g., extending an open palm up toward an interlocutor to give them the turn), affect displays (e.g., tense hand movements), and adaptors. Adaptors encompass self-manipulation movements like touching, rubbing, or scratching. These unconscious actions are thought to be devoid of overt communicative intent (Ekman and Friesen, 1969), thus distinguishing them from speech-gestures, although some theories propose that adaptors serve as strategic gestures to help speakers manage stress (Barroso et al., 1978; Harrigan et al., 1986; Mohiyeddini and Semple, 2013; Pang et al., 2022). Freedman (1972) introduced an additional subclassification of adaptors: discrete and continuous body-touching movements. Discrete adaptors are brief, lasting less than 3 s, typically directed toward the face or head, while continuous adaptors involve longer, repetitive actions like scratching or rubbing hands, also referred to as “fidgeting” (Mehrabian and Friedman, 1986).

McNeill, focusing on speech-gestures, placed them on a continuum depending on their relationship with speech (McNeill, 1992): Iconical gestures illustrate concrete semantic meaning while metaphorical gestures relate to abstract meanings; deictic gestures point to existing or abstract entities and beats stress parts of the utterance. Iconical and metaphorical gestures are labeled representational and, together with deictic gestures are referential (as they refer to the content of the utterance). Under Ekman and Friesen’s categorization they are all illustrators, together with beats—quick movements that stress parts of the utterance which often accompany prosody. However, many metaphorical gestures have primarily meta-discursive functions: interactive, flow management (discursive) or cognitive (illustrating inferences or the speaker’s stance), see Lopez-Ozieblo (2020) for a detailed description—some regulators would fall under this category. Together with beats, these metaphorical gestures carry discursive-pragmatic functions, enhancing interaction or managing the discourse.

**Figure 1:**
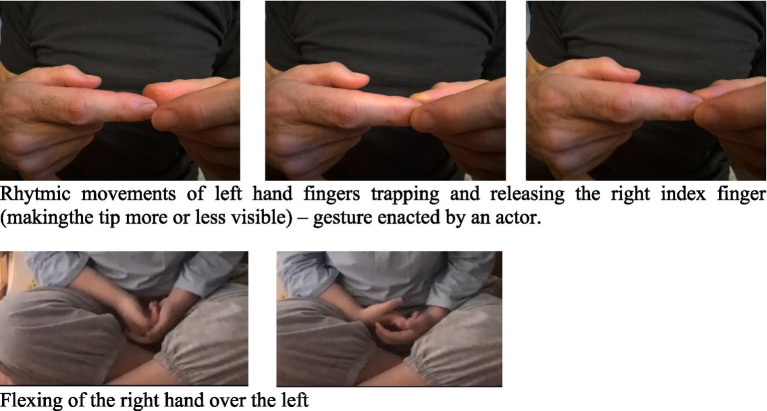
Examples of “flutters”.

The present study covered all hand-movements observed during the speech of our participants (see section 2.3 for details). In addition to adaptors, we also noted micro-gestures which we labeled as “flutters.” Flutters are often categorized as ‘other gestures’ and not included in studies (e.g., Hostetter and Hopkins, 2002; Kopple, 2014; Nagpal et al., 2011) or coded as beats (Gullberg, personal communication) or fidgets (Frances, 1979). These gestures do not fall in the same category as adaptors as they are somewhat controlled and correlate to the prosody of the utterance. Flutters seem to be speech-gestures that have been inhibited, at least to the extent of not resulting in a full gesture, but due to their micro-nature it is difficult to identify their form and thus to categorize their nature (e.g., a finger movement might be tracing a path or it could just be a beat), see Figure 1.

### 1.3 Personality and gestures

Gestures are cognitive and communicative resources (Gullberg, 2022) conveying semantic, interpersonal and social information (Nagels et al., 2015) which is processed by the interlocutor regardless of what is triggering the gesture. In L2 speakers the trigger might be cognitive (to help themselves think and produce the right words) or it might be that it is in the nature of the person to gesture. Kelly and Ngo Tran (2023) add that emotions also need to be taken into consideration in gesture production. On the other hand, gesture production might be personality driven. Previous studies on personality and gestures have focused on L1 speakers, reporting some relationships between gestures and personality traits, although—as with personality and proficiency—not all results have been replicated. We are not aware of any study specific to L2 speakers’ gestures and personalities.

Previous studies exploring the relationship between personality and gestures in L1 have employed a number of personality tests, BFI-44 (Canarslan and Chu, 2024; Hostetter and Potthoff, 2012; Li, 2023); Eysenck’s (Nagpal et al., 2011; O’Carroll et al., 2015); Eysenck and Catell’s (Campbell and Rushton, 1978); NEO-60 (Berry and Hansen, 2000; Kopple, 2014); NEO-PI-R (Cuñado Yuste, 2017), often resulting in conflicting results. Conflicts are more likely the result of the variation in task eliciting gestures and how gestures have been classified and measured. Studies measuring frequency of gestures over time (Kopple, 2014) ignore the fact that speech-gestures tend to co-occur with speech while many adaptors can be observed during speech pauses and their meaning is heavily linked to their duration. Continuous adaptors, such as scratching, and some flutters lend themselves to being counted not as instances but as the overall time spent scratching or finger tapping. Studies measuring frequency of gesture per word (Hostetter and Potthoff, 2012) might not accurately reflect the time spent on continuous gestures. In other studies, the total number of gestures observed is compared between individuals carrying out the same task, regardless of the time spent talking (Li, 2023), which might misrepresent the relationship between gesture and speech. In this study we annotated all finger and hand movements, calculating their frequency according to their nature.

In gesture-personality studies, the two most tested dimensions have been extraversion and neuroticism, often tested in clinical studies because of their links with anxiety. Neuroticism has been found to have positive correlations with a range of gestures including: iconical and metaphorical representational gestures (Hostetter and Potthoff, 2012); beats and metaphorical gestures (Kopple, 2014); and adaptors (Cuñado Yuste, 2017; Ekman and Friesen, 1972; Mehrabian and Friedman, 1986; Pang et al., 2022). However, Berry and Hansen (2000) reported a non-significant negative correlation with illustrators (representational gestures), Canarslan and Chu (2024) found no correlations with representative and deictic gestures and Li (2023), testing only deictics, found no correlation either.

Extraversion has been found to be positively correlated with representational gestures in general (Hostetter and Potthoff, 2012), although Canarslan and Chu (2024) did not find any; correlations were found between extraversion and iconical gestures specifically (O’Carroll et al., 2015) and with deictic gestures (Li, 2023). Berry and Hansen (2000) also found a non-significant positive correlation with illustrators. However, other studies did not find any correlations with any type of gesture including adaptors (Campbell and Rushton, 1978; Cuñado Yuste, 2017; Kopple, 2014; Nagpal et al., 2011).

Agreeableness has been found to be positively correlated with iconical gestures (Kopple, 2014) and illustrators and regulators (Cuñado Yuste, 2017). However, Berry and Hansen reported a negative correlation with illustrators. Cuñado Yuste (2017) also found positive correlations between illustrators and openness, and between regulators and conscientiousness, results which have not been confirmed by other studies. See Table 1 for a summary of these results, including the significant correlations.

**Table 1:** Summary of results from previous studies.

Authors	Study measurement instruments	Neuroticism	Extraversion	Agreeableness	Openness	Conscientiousness
Hostetter and Potthoff (2012)	Representational (iconical and metaphorical) gestures per log of words / BFI-44	+ve corr. With iconical and metaphorical representational	+ve corr. Representational	No corr.	No corr.	No corr.
Kopple (2014)	All except adaptors (gesture per minute) / NEO-60	+ve corr. Beats and metaphorical	No corr.	+ve corr. Iconical and -ve non-significant with beats and metaphorical	No corr.	No corr.
O’Carroll et al. (2015)	Representational (iconical + metaphorical) and deictic / Eysenck	NA	+ve corr. With iconical gestures	NA	NA	NA
Canarslan and Chu, 2024	Representational (iconical + metaphorical) and deictic / BFI-44	No corr.	No corr.	No corr.	No corr.	No corr.
Mehrabian and Friedman (1986)	Fidgeting (self-reported via questionnaire)	+ve corr. Fidgeting	NA	NA	NA	NA
Cuñado Yuste (2017)	Illustrators, regulators and adaptors (not clear how gesture rate measured) / NEO-PI-R	+ve corr. Adaptors	No corr.	+ve corr. Illustrators and regulators	+ve corr. Illustrators	+ve corr. With regulators
Ekman and Friesen (1972)	Adaptors (rates not clear)	+ve corr. Adaptors	NA	NA	NA	NA
Campbell and Rushton (1978)	All-gestures and adaptors / Eysenck and Catell	No corr.	No corr.	NA	NA	NA
Pang et al. (2022)	Adaptors (self touch time/narration time) / BFI-44	+ve corr. Adaptors, neuroticism, state and trait anxiety. In multiple regression only state anxiety predicted adaptors.	No corr.	-ve corr. With adaptors (not significant in model their).	No corr.	No corr.
Berry and Hansen (2000)	Gestures that illustrate the verbal content (total number of gestures in dyads talking 6 min) / Neo-60	Non-significant - ve corr. Illustrators	non-significant +ve corr. Illustrators	-ve corr. Illustrators	non-significant - ve corr. Illustrators	non-significant +ve corr. Illustrators
Nagpal et al. (2011)	Bilinguals. Iconical and deictic + conventional (semi-emblems) per 100 words / Eysenck	NA	No corr.	NA	NA	NA
Li (2023)	Deictic gestures (manual and others) / BFI-44	No corr.	+ve corr. Deictic (manual)	No corr.	No corr.	No corr.
Chu et al. (2014)	Representational (iconical + metaphorical + deictic), / Empathy Quotient	NA	NA	+ve corr. Discursive	NA	NA

All of these studies focus on Western participants, leaving an important gap in our understanding of the relationship between personality and gesture. As gestures are linked to both speech and thought (McNeill, 2005), culture also affects how individuals gesture, based on: variations in spatial and time reference frames used; linguistic structures; or pragmatical content load (Kita, 2009). These differences, some of which are transferred from the L1 to the L2, result in cultures which are more likely to gesture than others, such as American English vs. Chinese Mandarin speakers (So, 2010). Therefore, culture should also be a variable to consider in personality-gesture studies.

### 1.4 Proficiency and gestures

Gestures in bilinguals (defined as individuals who know and use more than one language to communicate, irrespective of their level of fluency in those languages; Grosjean, 2010) have been extensively studied, concluding that bilinguals gesture more than monolinguals, with a noticeable difference particularly in illustrators and iconical gestures (Nagpal et al., 2011; Nicoladis, 2007; Pika et al., 2006; So, 2010), although the frequency of gestures is usually the same in both languages if they are spoken with similar proficiency (Sherman and Nicoladis, 2004). The frequency of different types of gesture have been noted to vary by proficiency (Gullberg, 1998; Lopez-Ozieblo, 2022), with more iconical gestures observed in speakers when proficiency is higher (Gregersen et al., 2009; Lin, 2019). Lin (2019), studying Chinese speakers of English, also observed more beats in higher proficiency speakers and more deictics in lower proficiency individuals. However, Gol and Aminzadeh (2015), observing Iranian speakers of English, noted more deictic and iconical gestures in intermediate rather than higher proficiency speakers, as did Varnosfadrani and Tavakol (2022). Nicoladis et al. (2007) posited that gesture rates were likely to be influenced by the task itself rather than proficiency. However, Aziz and Nicoladis (2019) did not find task or proficiency to impact gesture. One possible explanation for these conflicting results, the one we explore in this study, is that gestures in the second language could also be affected by the second language personality of the speakers, or other variables such as gender or age.

### 1.5 Gender and gestures

The possible effects of gender on gesture are not well understood. Gender differences in gesture production have been observed in infants, with girls producing a wider variety of gestures than boys (Germain et al., 2022). In adults, earlier studies had observed that women produced more speech-gestures than men, being more expressive (Frances, 1979), although the number of iconical gestures was the same (Hostetter and Hopkins, 2002) but men were reported to be more fidgety and restless (Briton and Hall, 1995; Hall, 1990) and to produce more adaptors (Skomroch et al., 2013). Yang (2010) reported that among Mandarin Chinese speakers, women produced specific gestures, like hand-shielding-mouth gestures when laughing and hand clapping when excited, while men produced more index-finger pointing for indicating targets and drawing attention. In studies with bilingual speakers, women have been found to produce longer narratives with more referring expressions and gestures in their second language but this was affected by proficiency as well (Nicoladis et al., 2007; Varnosfadrani and Tavakol, 2022). As gender might be a variable correlated to gesture production and personality, it was also included in this study.

## 2 The study

Our hypothesis was that personality would be affecting the gestures of the L2 speakers who participated in this study, perhaps enough to mask the effects of proficiency. Based on previous studies of personality and gestures, and personality and L2 communication, we expected to find that extraverts are more fluent in their speech (including higher speech rates) and also produce more speech-gestures than other speakers due to higher energy levels (Hostetter and Potthoff, 2012); but not more adaptors or flutters, as these individuals tend to have higher self-confidence levels (Dewaele, 2012; leading to fewer adaptors) and their gestures will be produced in full (resulting in fewer flutters). Individuals with higher levels of neuroticism were expected to produce more speech-gestures synchronous with the speech to improve their narrations (Robinson et al., 1994), but as they might also be more anxious than others (Dewaele, 2002), higher levels of adaptors and flutters were expected. We suspected that more open individuals (more creative, innovative, open to ideas) might use more semantic gestures than those less open, reflecting a link between creativeness and gesture (as suggested by Prof. Gale Stam during a personal exchange). Conscientiousness might be correlated with a preference for discursive gestures, including those used to ‘order’ the elements of an utterance and its prosody, to add precision to the narration (Ehrman, 2008). More agreeable individuals, with higher levels of empathy, might produce more speech-gestures (Kopple, 2014) to better relate with their interlocutor (Nagels et al., 2015). In addition, we believed there might be a positive correlation between proficiency and semantic gestures (Gregersen et al., 2009; Lin, 2019) as proficiency develops the speech-gesture-thought link manifested in better synchronous speech-gesture production (Lopez-Ozieblo, 2022); and a negative one with discursive gestures (Lin, 2019), which might be more necessary to lower proficiency speakers who find it difficult to provide cohesiveness to narrations just with speech (Lopez-Ozieblo, 2022). Differences in gender might be reflected in more adaptors in men (Skomroch et al., 2013) and more semantic gestures in women (Frances, 1979), as observed in previous studies.

### 2.1 Methodology

This study used part of an existing gesture corpus collected over 3 years from sessions with over 100 Cantonese mother tongue speakers whose second language was English. Participants were university students or recent graduates improving or planning to continue improving their proficiency in English. The corpus for this study focused on the data from the first session collected from participants who were asked to watch and retell one of the *Tweety and Sylvester* TV cartoon episodes (Freleng, 1950). Their narrations were analyzed for gestures. Participants were then asked to complete the BFI. All interactions were carried out online.

### 2.2 Participants

Participants answered a call to participate in a 3 year-long study. Sessions were conducted every 6 months but for this study we collected data from the first session. This ensured that the novelty factor was the same for all participants, in terms of the task procedure, the contents of the narration and the relationship with the researcher. The first session was attended by 98 online participants. Thirteen were excluded as their L1 was found not to be Cantonese. Out of the remaining 85, 64 completed the BFI survey, three participants were subsequently excluded as they were over 35 when they started the study, this was done to homogenize the sample as much as possible. The remaining 61 participants were aged 18 to 32, with an average age of 21.2, 36% were male. Participants were all Cantonese mother tongue speakers who had started to learn English at kindergarten or shortly after. Some had attended English medium of instruction schools and others Cantonese schools. They were all either university students or recent graduates from higher institutions in Hong Kong. Participants’ oral proficiency was evaluated as part of the study by three independent professional proficiency evaluators engaged under this project, all experienced with IELTS testing and the Hong Kong context.

Participants were told the study explored their communicative behaviors but no mention of gestures was made until after the last data recording sessions, when debriefing interviews were carried out to explain the purpose of the study. Two of the participants reported that they had guessed gestures might be part of the study, as all sessions were videorecorded, but they all thought the main focus was oral proficiency. Participants’ consent was obtained at the beginning of the main project and again when completing the personality questionnaire and at the end of the project. Participants were paid after each session attended starting from the second one.

### 2.3 Procedure

As the main project began during the COVID pandemic, it was necessary to carry out the study online. Space and privacy in Hong Kong contexts can be an issue, therefore we asked participants to sit on their beds cross-legged (a few sat on the floor) about 1.5 meters from the camera which ensured we could capture most of their bodies. All methods were carried out in accordance with relevant guidelines and regulations, protocols were approved by the ethics committee of the Hong Kong Polytechnic University and informed consent was obtained from all subjects.

Sessions with participants were led by the author (except on 5 occasions where a second researcher led the sessions), the interlocutors were visible on video throughout the session. Sessions were carried out via Zoom and recorded, and lasted about an hour. Participants were asked to watch half an episode of a *Tweety and Sylvester* TV cartoon episode. This replicated well-tested input used in many studies (Brown and Gullberg, 2008; Kita and Özyürek, 2003; McNeill, 2005). The participants were told to watch the cartoon first and then narrate it in English in as much detail as possible. They were not allowed to take or use notes when narrating the story. The interlocutor was visible on their screens but aside from smiling and nodding no other feedback or prompts were given.

#### 2.3.1 BFI

Participants completed the BFI online about 6 months after the first session, ensuring that their answers were not associated with that session. Results of BFIs are fairly stable over time (with some known long-term related changes; Chen et al., 2022; Terracciano et al., 2006). The questionnaire was adapted from the simplified Chinese characters version from John’s lab (John et al., 1991) and converted to traditional characters given the Hong Kong context (no changes were made to content).

After collecting and analyzing the data it was noted that a confounding factor, and a limitation of the study, was that, in bilingual speakers, answers to the BFI can vary by language (Dylman and Zakrisson, 2023), as noted above. Although our participants were not fully bilingual it is possible that their “second language (L2) personality” might differ from their “first language (L1) personality.” Therefore, about 18 months later after the Chinese BFI was completed, we asked participants to redo the BFI, this time in English, again using the version by John et al. (1991). Thirty-nine participants completed it, which gave us enough data to compare the results from the English and Cantonese versions. Paired sample t-tests confirmed that there were no significant differences in any of the dimensions and the effect sizes were low. Therefore, we carried out subsequent analyses using the Chinese BFI data for the 61 participants. The self-reported answers for each of the personality dimensions were tested for internal consistency using Cronbach’s alpha and the results were found to range between 0.702, for agreeableness and openness and 0.834 for extraversion (where 0.7–0.79 is acceptable; 0.8–0.89 is good; Barker et al., 1994).

The English BFI coincided with a post-study feedback session with the participants where we discussed with them their use of gestures. During this session, some of the participants’ comments led us to believe that age might have had an effect on their gestures and this variable was also added (see the Discussion section).

#### 2.3.2 Gestures

Gestures produced during the narration were transcribed together with the speech. The narrations were first transcribed into speech using PRAAT and the gesture analysis was carried out in ELAN. Three annotators carried out the transcriptions and these were checked by two additional gesture annotators who, working independently, transcribed the gestures in ELAN, with a third transcribing 70% of the data. Disagreements were discussed between the first two annotators achieving a preliminary inter-rater agreement of over 90% and after discussion, complete agreement was reached on the classification of the gestures.

Previous personality-gestures studies have used both Ekman and Friesen and McNeill’s categorizations, although not always covering all types of gestures. In this study we included all gestures: speech-gestures as well as adaptors and micro-gestures or “flutters.” McNeill stressed that most gestures display attributes that overlap two or more of the continuum categories (2005). Thus, in this study, we categorized gestures by their primary nature. If a gesture was both illustrating a concept and stressing it, the former function was noted.

Our categorization included: all-gestures, which were then subdivided into speech-gestures, adaptors and flutters. Flutters were categorized separately as they are ambiguous micro-movement of the fingers or hands, often not visible in the first pass, which we could not associate with a specific function but were clearly integrated with the prosody of the utterance, see Figure 1. If this synchronicity was not observable the gesture was labeled an adaptor (such as scratching an arm or rubbing the hands together). Speech-gestures were further subdivided by their primary function, either referential-semantic (labeled “semantic”) or discursive-pragmatic (labeled “discursive”). The more commonly used “referential” label includes all metaphorical gestures, many of which have discursive rather than semantic functions. Semantic gestures included representational iconical and metaphorical gestures that referred to the semantic content of the speech, as well as deictics (pointing) and emblems (codified gestures which might not require speech to be understood, such as a bye-bye wave. We did not observe many of these in our study). Discursive gestures included beats – often used to mark prosody or to separate and place in space the various linguistic elements of the utterance –, as well as other metaphorical gestures, such as circling the wrist to indicate ‘again’. When necessary, the content of the speech was used to disambiguate the category of the gesture. For example, a sweeping away gesture from the negating family (Bressem and Müller, 2017) occurring with a linguistic negative marker would have been recorded as a semantic gesture, while the same gesture occurring without the referent but adding a pragmatic meaning of dismissal, would have been recorded as discursive.

For the number of words, we included fillers such as *eh*, *ehm* and cut-offs (interrupted words). Interrupted gestures were also counted, these were often subject to discussion. Cyclic gestures (e.g., a hand turning at the wrist twice accompanying the utterance “again and again”) were counted only once, other repetitions were counted as separate instances, as were repeated words. Gesture rates were calculated per 100 words to facilitate reading of the data (following Hostetter and Potthoff, 2012; Nagpal et al., 2011; Nicoladis et al., 2007). The frequency of adaptors was also calculated per 100 words. Some flutters and adaptors (those labeled continuous by Freedman, 1972) are often repetitive in nature and if we had counted them as one gesture we might have misrepresented the full extent of their co-occurrence with speech or pauses. Therefore, it was decided to calculate the duration of flutters and adaptors over the total narration time to provide a more accurate reflection of their occurrence in the narration. All times are reported in minutes to follow previous studies (e.g., Kopple, 2014).

#### 2.3.3 Proficiency

Proficiency was evaluated by three independent certified proficiency evaluators, all with Hong Kong experience in oral proficiency testing of English as a second language. Oral proficiency was evaluated based on the video recordings of the narrations and evaluated following a multi-level proficiency scale, based on the Common European Framework of Reference for Languages (Council of Europe, Council for Cultural Co-operation, Education Committee, Modern Languages Division, 2001) and the Cambridge scales, that allowed evaluators to score from A1 to C2 levels using a 1 to 120 scale. Parity checks were carried out on 10% of the data and the Facets model, also known as the Many-Facet Rasch Model (MFRM), an extension of the Rasch model (Myford and Wolfe, 2003), was used to analyze the consistency and fairness of the scoring across different examiners, following the practice of the Cambridge First oral exams. Facets calculations for inter-rater and intra-rater consistencies showed these to be above 85% in all cases [the Rasch equivalent of Cronbach’s alpha; Linacre (n.d.)]. The average proficiency level was 85, on a scale of 1 to 120, which corresponds to a low C1 level, with the proficiency range varying between 63 (low B2) and 113 (low C2).

### 2.4 Analysis

After analyzing each of the variables, it was found that a number of the distributions was not normal, there were various issues including skewedness and high variances. Therefore, whenever necessary, non-parametric tests were employed. These included Mann–Whitney tests to compare the data by gender, and Kendall’s tau-b correlations to obtain a wholistic picture of potential correlations between all the variables. A series of Gaussian generalized linear regression models were then run to measure the relationship between each type of gesture, (the dependent variable) and the personality dimensions, age, gender and proficiency (independent variables).

Based on the literature review, it was suspected that some of the independent variables might be correlated, in particular some of the personality ones, such as neuroticism and extraversion and also proficiency and extraversion. Therefore, we carried out a correlation analysis with the independent variables to identify strong correlations that might lead to multicollinearity issues in the generalized linear regression. As some of the variables were not normal distributions and the sample sizes were relatively small, a Kendall tau-b correlation was carried out, adjusting for the multiple comparisons by using Bonferroni, to account for Type I errors. There were eight variables (the five personality traits, gender, age and proficiency), leading to 28 possible comparisons. Dividing the significance level 0.05 by 28 resulted in a new significance level of *p* = 0.00179. Although this is a very conservative correction, a strong significant correlation was found between extraversion and neuroticism *Kendall’s tau B* = 0.412, *p* < 0.001, a medium-high relationship coefficient, with a medium-high effect size Fisher’s *z* = 0.439, (0.5 is considered a high effect, Cohen, 1988, Corder and Foreman, 2014). Two other possible correlations were also identified, between neuroticism and agreeableness and openness and extraversion, although in both cases the *Kendall’s tau-B* was below 3 and *p* values above the threshold. See Footnote 1 for all calculations.

To avoid multicollinearity issues two sets of models were generated, one excluding extraversion (model 1) and the other excluding neuroticism (model 2), this also accounted for the possible relationships between neuroticism and agreeableness and openness and extraversion. Deviance goodness-of-fit test was checked for the fit of the model and multicollinearity was tested by checking tolerance and VIF.

For all effect sizes we used Cohen’s interpretation: 0.10: small effect; 0.30: moderate effect, 0.50: large effect (Cohen, 1988). The R square values of the models were interpreted as small if 0.01 or below, indicating that the model explains very little of the variability in the data; moderate if between 0.09 and 0.25; and high if 0.25 or above 0.25 (Kutner et al., 2005).

All statistical tests and modeling were carried out using the statistical program JASP (JASP Team, 2023). Based on the five fixed effects of the BFI and at least two random effects (e.g., participant and proficiency), a power analysis using G*Power (Faul et al., 2009) had indicated that a minimum sample size of 63 participants (α error: 0.05) would be necessary. Note that we stretched the boundary by proceeding with 61 participants.

## 3 Results

Overall, we recorded and annotated 4,075 gestures in 61 participants producing a total of 21,387 words during 166 min of narrations. The average speech rate was 129.91 words per minute and the average narration time was 2.7 min. The total time spent gesturing, including flutters, was 129.32 min. The average participant gestured during 78.5% of the narration time (2.12 min). All individuals were found to gesture at least four times or more, the average being 67 gestures, during varying lengths of narrations. For detailed descriptive statistics (See Footnote 1).

The average frequency of all-gestures per 100 words was 20.07, this includes adaptors and flutters. Most of these were speech-gestures (*M* = 16.3, *SD* = 1.05) with a prevalence of pragmatic-discursive gestures (*M* = 10.769, *SD* = 0.805).

### 3.1 Proficiency

Proficiency was found to have negative relationships with the frequency of all-gestures, speech-gestures and discursive gestures when analyzed per 100 words. The relationship with semantic gestures per 100 words was positive, as predicted. Adaptor and flutter duration per narration time all had a negative relationship with proficiency. However, none of these relationships was strong or significant with *tau-b* and *z* values all below 0.15, although they confirm previously reported trends.

### 3.2 Gender

A total of 22 men and 39 women completed the first session and the BFI. Women talked slightly longer than men (*M_women_* = 2.759, *SD_women_* = 0.19; *M_men_* = 2.656 *SD_men_* = 0.253). As a ratio, gesture time over narration time, women gestured slightly more than men (*M_women_* = 0.797, *SD_women_* = 0.048; *M_men_* = 0.765 *SD_men_* = 0.006) but also spoke faster, in terms of words per minute (*M_women_* = 133.757, *SD_women_* = 5.202; *M_men_* = 123.092 *SD_men_* = 4.097). However, none of these differences was found to be significant. The effects sizes were found to be below 0.3 in all cases, therefore low.

The frequency of all-gestures per 100 words was slightly higher in women than in men (*M_women_* = 20.355, *SD_women_* = 1.318; *M_men_* = 19.572 *SD_men_* = 1.971). Frequency of all types of speech-gesture per 100 words was also higher in women than in men. However, men’s duration of adaptors and flutters per narration time was higher than women’s. None of these differences was found to be statistically significant and the effects were low, but they confirm the trends found in previous studies.

In terms of personality, women were found to report higher levels of agreeableness, conscientiousness and neuroticism, while men reported higher levels of openness and extraversion. Statistically, none of these differences was significant although neuroticism did indicate a close to moderate effect (*M_women_* = 3.497, *SD_women_* = 0.104; *M_men_* = 3.188 *SD_men_* = 0.137 *t*(59) = −1.798, *p* = 0.077, Cohen’s *d* = −0.479 *CI* [−1.007, 0.052]).

Women’s proficiency levels were found to be slightly higher than men’s (*M_women_* = 85.215, *SD_women_* = 1.498; *M_men_* = 84.57 *SD_men_* = 1.984) but these differences were not statistically significant and the effect was low.

### 3.3 Correlations between variables

A series of generalized linear regression models were run to confirm the combined effects of the variables tested on gesture production. Each type of gesture was modeled with one set of models that excluded extraversion (model 1) and another set excluding neuroticism (model 2), as mentioned in the analysis section. Significant results are given in Table 2, for the full set of results.^1^

**Table 2:** Coefficients with significant relationships found in the generalized linear regression models.

Model 1 excluding extraversion		Deviance	*p*	*AIC*	*BIC*	*df*	*Χ^2^*	*p*	Coefficient	*Estimate*	*SE*	*t*	*p*	Confidence interval	
Frequency of all-gestures per 100 words	H₀	4376.94		437.778	441.999	60									
	H₁	3778.66	< 0.001	442.812	461.81	53	598.279	0.32	Agreeableness	4.568	2.221	2.057	0.045*	0.215	8.921
Frequency of speech-gestures per 100 words	H₀	4048.31		433.016	437.238	60									
	H₁	3642.24	< 0.001	440.569	459.567	53	406.071	0.556							
Frequency of semantic gestures per 100 words	H₀	848.914		337.729	341.95	60									
	H₁	710.836	< 0.001	340.9	359.898	53	138.078	0.198	Agreeableness	2.46	0.963	2.553	0.014*	0.572	4.348
Frequency of discursive gestures per 100 words	H₀	2371.18		400.387	404.609	60									
	H₁	2170.76	< 0.001	409	427.998	53	200.414	0.673							
Frequency of adaptors per 100 words	H₀	107.653		211.761	215.983	60									
	H₁	95.439	< 0.001	218.415	237.413	53	12.214	0.463							
Adaptor time per narration time	H₀	0.331		−141.02	−136.8	60									
	H₁	0.284	1	−136.41	−117.41	53	0.047	0.288							
Flutter time per narration time	H₀	1.541		−47.259	−43.037	60									
	H₁	1.147	1	−51.284	−32.286	53	0.394	0.022*	Age	−0.015	0.006	−2.675	0.01*	−0.03	−0.004
									Neuroticism	−0.078	0.034	−2.306	0.025*	−0.14	−0.012
Model 2 excluding Neuroticism		Deviance	*p*	*AIC*	*BIC*	*df*	*Χ^2^*	*p*	Coefficient	*Estimate*	*SE*	*t*	*p*	Confidence interval	
Frequency of all-gestures per 100 words	H₀	4376.94		437.778	441.999	60									
	H₁	3623.03	< 0.001	440.246	459.244	53	753.912	0.163	Agreeableness	4.56	1.994	2.287	0.026*	0.651	8.468
Frequency of speech- gestures per 100 words	H₀	4048.31		433.016	437.238	60									
	H₁	3508.41	< 0.001	438.285	457.283	53	539.9	0.338							
Frequency of semantic gestures per 100 words	H₀	848.914		337.729	341.95	60									
	H₁	702.831	< 0.001	340.209	359.207	53	146.083	0.164	Agreeableness	2.184	0.878	2.486	0.016*	0.462	3.905
Frequency of discursive gestures per 100 words	H₀	2371.18		400.387	404.609	60									
	H₁	2104.27	< 0.001	407.102	426.1	53	266.91	0.469							
Frequency of adaptors per 100 words	H₀	107.653		211.761	215.983	60									
	H₁	96.515	< 0.001	219.099	238.097	53	11.138	0.533							
Adaptor time per narration time	H₀	0.331		−141.02	−136.8	60									
	H₁	0.278	1	−137.78	−118.79	53	0.054	0.201							
Flutter time per narration time	H₀	1.541		−47.259	−43.037	60									
	H₁	1.237	1	−46.661	−27.663	53	0.304	0.095	Age	−0.016	0.006	−2.822	0.007**	−0.03	−0.005

*p < 0.05, **p < 0.01.

The Extraversion model excludes the variable neuroticism and the Neuroticism model excludes extraversion.

The results suggest that variables other than the ones considered are affecting gesture production. In both models, agreeableness is the only personality variable related to speech-gestures. Agreeableness is positively related to semantic gestures, the more agreeable the individual is the more they gesture. The other significant correlations found were with flutters, which have not been covered by previous studies. Flutters were negatively correlated to age, in both models, and also to neuroticism. Extraversion, which we expected to be a significant factor affecting gestures, was not found to be so. Neither proficiency nor gender were found to affect any type of gesture.

For all dependent variables, both sets of models show deviances for the null hypotheses, H_0_, greater than for the proposed model, H_1_, indicating that the proposed H_1_ model is a better fit than the null model, so the various variables tested do have an effect on the gesture dependent variable. However, in most cases *p* < 0.001 for deviance differences, suggesting the models are still not very good fits. The only exception is when modeling adaptors and flutter duration over narration time, where *p* = 1, indicating a strong goodness of fit. In most cases the *p* value for Chi square is above 0.05, confirming that the independent variables tested do not fully explain the variation in the dependent variables.

## 4 Discussion

Many of our expectations were confirmed, in terms of whether the relationships were positive or negative, but these were seldom found to be significant or with high effect levels. In this group of participants, the only significant correlations found were a positive one between agreeableness and semantic gestures; and negative ones between age and flutters; and neuroticism and flutters. The effects of all three were low to moderate. Contrary to what has been previously reported, we did not find significant relationships between neuroticism and adaptors or semantic gestures, nor between extraversion or openness and semantic gestures. Proficiency and gender had little effect on gestures. These are unexpected results that suggest that other factors, rather than the variables tested, are affecting speech production. We suggest that these factors are related to specific emotions and emotional constructs, such as anxiety, self-consciousness and empathy, which are moderating the effects of personality.

In addition to the three significant correlations found, the following relationships were also observed, confirming our expectations: positive correlations between conscientiousness and discursive gestures and agreeableness and speech-gestures in general, neuroticism and speech-gestures. We also found proficiency to have a positive relationship with semantic gestures and a negative one with discursive ones. Contrary to our expectations, openness and semantic gestures were negatively correlated as were extraversion and speech-gestures but adaptors and flutters had a positive correlation with extraversion. Men were more likely to produce adaptors than women, while women produced more semantic gestures. Age was found to have a significant negative correlation with flutters.

### 4.1 Proficiency

The most striking result was the lack of significant correlations between proficiency and any type of speech gesture per 100 words. However, although not significant, we were able to confirm the previously reported positive relationship between proficiency and semantic gestures (Gregersen et al., 2009) and a negative one with discursive gestures (Lin, 2019). Adaptors and flutters, on the other hand, decreased with proficiency, again, these results were not significant.

The lack of significant results related to proficiency might have various explanations: It is possible that the range of proficiencies among our participants was too narrow to reflect strong correlations with gestures. Another possibility is that as our evaluation of proficiency was restricted to oral narration skills, unlike other studies which are based on written proficiency tests or more wholistic oral proficiency skills, we failed to capture proficiency correctly and this affected results. However, the more likely explanation is that there are other variables impacting gesture production which weigh more heavily than proficiency (Gullberg, 2022). Some of these have been suggested in previous studies and include: cognitive traits such as memory skills (Chu et al., 2014); the task itself (Lin, 2019); individual styles (Nagpal et al., 2011); anxiety (Gregersen, 2005) or the relationship with the interlocutor (Brown et al., 2022), which could be related to emotional factors. Specifically, empathy and anxiety might affect willingness to communicate (Oxford, 2016) as well as modulate how that communication takes place, triggering semantic gestures to ensure mutual understanding (Nagels et al., 2015) or adaptors and flutters potentially controlling anxiety (Barroso et al., 1978; Harrigan et al., 1986; Mohiyeddini and Semple, 2013; Pang et al., 2022). In this study we also need to include as potential factors the new (at the time) online environment, that the participants might not have been familiar with, and the relatively low level of interaction with the interlocutor.

### 4.2 Gender

Gender, contrary to results from previous studies (Hall, 1990; LaFrance and Vial, 2016), showed no significant correlation with speech-gestures. However, the overall trends previously reported were confirmed, as men were found to produce more adaptors than women (Skomroch et al., 2013) and women were found to produce more speech-gestures than men (Frances, 1979). In our study, although there were some small differences in personality by gender, these were not significant. A previous study with 201 Hong Kong residents (Schmitt et al., 2008) had noted slightly higher levels of extraversion in men than women and our results confirmed this. All other dimensions were higher in women, including openness, previously reported as higher in men (Schmitt et al., 2008). One point to note is that we had a relatively small group of participants and a majority of women and this might have affected the results, future studies should seek a more balanced gender ratio as well as a larger sample.

### 4.3 Personality

Most of our predicted relationships were met, however, only one was found to be significant and two unexpected ones with flutters were also found. The only significant relationship found between speech-gestures and personality was with agreeableness. Flutters were found to be negatively correlated with neuroticism and age.

#### 4.3.1 Agreeableness

Our study found positive significant correlations between agreeableness and the frequency of all-gestures per 100 words. This relationship is possibly caused by the significant positive correlation between agreeableness and semantic gestures specifically, as agreeableness is not observed to affect other types of gesture. These results corroborate those of Cuñado Yuste (2017), who had reported a positive correlation with illustrators (a type of semantic gesture) and of Kopple (2014) who found a strong positive correlation between agreeableness and iconical gestures.

Hostetter and Potthoff (2012) had expected, but did not find, any correlations with agreeableness and gesture rate. They speculated whether this might have been related to the monologic nature of the task which would minimize cooperation. Our task was also a monolog but the interlocutor was clearly listening, perhaps increasing the level of interaction vs. Hostetter and Potthoff’s task. Our participants were also keen to do their best (as reported in the exit interviews). Agreeableness is related to empathy and also to being cooperative and compliant (Costa and McCrae, 1992a) which might have translated as a higher level of engagement with the task, leading to more detailed narrations and an effort to convey meaning accurately through gestures. Agreeableness might be augmented in this academic Chinese context, where the teacher/researcher is an authority to be respected and their instructions to be followed (Jin and Cortazzi, 2006) more so than in Western contexts Littlewood (2001).

#### 4.3.2 Conscientiousness

The expected positive correlation between conscientiousness and discursive gestures, was observed but proved not to be significant. Conscientious individuals are also deliberate and organized (Ehrman, 2008), which might lead to L2 speakers producing more structured narrations with discursive markers and gestures to provide the organization flow to the narration. A detailed analysis of the utterances of these individuals, looking at the structure of their narrations, would help to further explore this hypothesis.

#### 4.3.3 Openness

We had expected higher levels of openness to result in an increase in semantic gestures. As openness is linked to creativity and imagination we predicted that gestures could be an expression of that creativity but the relationship was found to be a negative one. The task was based on an existing story, which would not have given participants much room to be creative. As most studies of this nature do not offer many chances for participants to be creative [e.g., Hostetter and Potthoff’s in 2012 asked participants to define words; Kopple (2014) to retell stories], future studies could explore adding a story-creating task, rather than one where a story is repeated.

#### 4.3.4 Extraversion

Based on the results of previous studies (Hostetter and Potthoff, 2012; O’Carroll et al., 2015) we expected to find significant positive correlations between extraversion and all types of speech-gestures, specifically semantic ones, and a negative one with adaptors. However, the relationships found were the reverse although none were significant and the effects were on the low side of moderate for speech-gestures and very low for adaptors and flutters.

It is possible that our results did not find significant correlations between gestures and extraversion as more than half of our participants were female (64%), whose extraversion levels are lower than those of men. We think this is unlikely and offer an alternative observation based on the comments from participants: A number of individuals reported that at school and university they had been taught to gesture when presenting and talking in English. They also believed, based on TED talks and similar videos on the internet, that gestures could help them seem more assertive and clearer in their communication. Thus, a learnt social behavior might be overriding participants’ natural tendency to gesture.

#### 4.3.5 Neuroticism

Neuroticism was expected to lead to more speech-gestures, adaptors and flutters. Contrary to our expectations, we found a significant, although low effect, negative correlation between neuroticism and flutter time. The relationship with adaptors was also negative but not significant, while those with the various types of speech-gestures were positive, the effects high but not significant, confirming Hostetter and Potthoff (2012) results.

Hostetter and Potthoff (2012) reported an unexpected positive correlation between neuroticism and representational gestures (in our study, under the semantic category)—contradicting previous studies where no correlations were found (Borkenau and Liebler, 1992; Campbell and Rushton, 1978). They tentatively associated their results to the nature of the task required by their study. As Dewaele (2012) noted, different tasks bring out different personality traits. However, we would like to suggest that a positive relationship between neuroticism and speech-gestures in general might be likely in L2 speakers who are trying to perform well in oral tasks (Robinson et al., 1994), using gestures to excel in the communicative objective. At the same time they might also be experiencing more anxiety (Dewaele, 2002) but are controlling it, leading to fewer adaptors and flutters. Our participants were young adults, likely to be quite self-conscious, a trait also related to neuroticism. To try to appear less anxious, participants might have tried to control their gesture production, leading to fewer flutters and adaptors as they tried to appear more confident (this was mentioned in the exit interviews).

### 4.4 Age

Flutters were also found to have a moderate negative correlation with age, suggesting that older participants fluttered less than younger ones. There was an age range of 15 years among participants. Some were in their first year of university while others were postgraduates or in the workforce. Those in the workforce, even with only a few months of experience, generally displayed greater confidence and a more relaxed demeanor. Exit interviews revealed that most younger participants felt more nervous during the initial session, whereas many older participants reported little change in their anxiety levels. This suggests that the relationship with age may be superficial, warranting further exploration of confidence, which is linked to extraversion (Costa and McCrae, 1992a) and primarily associated with self-esteem and self-consciousness – traits related to neuroticism.

### 4.5 Other potential variables and limitations

There are a number of reasons why our study might not have found significant relationships. To start with, our categorization is a functional one; it is possible that we have erred in creating a functional semantic, discursive and adaptors categorization that is too broad. Further research is underway focusing specifically on adaptors, which might further clarify the role of these movements in the communicative act. For example, discrete adaptors, such as bringing a hand to the lips/chin are likely to occur with a cognitive pause and will be read by the interlocutor as a “thinking gesture.” These gestures have an important pragmatic function within the interaction, indicating a word-searching process (Skogmyr Marian and Pekarek Doehler, 2022). On the other hand, other adaptors, such as rubbing an arm, were sometimes noted to follow the prosodic patterns of the speech and might perhaps be stressing specific words or syllables. Leg, head and body movements which fell outside the scope of this study are also coordinated with gestures and speech and often carry pragmatic and deictic functions which we did not take into account.

The present study was based on tasks carried out online, which deviates from the face-to-face approaches utilized in previous research. This methodological distinction may contribute to the moderation of the relationships found resulting in no significant effects, contrary to the results of prior investigations.

Our participants were all Cantonese speakers and the study was linked to second language learning context. Therefore, it is possible that the differences observed between ours and previous studies, focusing on Western participants, might be related to cultural differences. Littlewood (2001) identified some small, but significant, differences in the behaviors and preferences of Western and Eastern (various countries) learners which might be affecting gesture production, such as anxiety when having to talk in the L2 class – reported as higher in Hong Kong learners than European ones. Culturally, there are also differences in gestures (Kita, 2009). Chinese monolingual speakers have been found to gesture less than American English speakers, although the differences are not so marked in bilinguals, suggesting there are low- and high-gesture cultures (So, 2010).

To enhance the generalizability and applicability of these findings, it is imperative to conduct further studies involving participants from diverse cultural and geographical backgrounds. This will ensure a more comprehensive understanding of the phenomena under investigation and address potential cultural biases inherent in the existing literature. The relatively modest sample size of 61 participants also limits the generalizability of the findings. A larger sample would potentially enhance the statistical power and robustness of the results. Another interesting question is whether these results will be replicated in a corpus of the mother tongue of our speakers (ongoing study).

## 5 Conclusion

In this study we explored possible relationships between individual variables, personality proficiency, gender and age and the frequency of various types of gestures, including adaptors and flutters. Overall, we conclude that there are other factors aside from the ones tested affecting gestures production in our participants, second language speakers of English. These factors are likely to be emotions and emotion-related constructs, such as anxiety, self-awareness, empathy or confidence, as well as learnt behaviors.

Although many of our expected personality-gesture relationships were noted, the only significant correlations we observed were a positive one between agreeableness and semantic gestures per 100 words; and negative ones between flutters and neuroticism and flutters and age. However, these relationships were at most moderate, confirming that factors other than the variables tested play a more important role in predicting gesture frequency.

Despite being mostly unconscious, gestures are influenced by society and the education the individual is exposed to. Gestures produced in formal contexts, such as the one in this study, are likely to be affected by previous educational experiences, like comments by language teachers or preparations to pass specific language exams. Participants believed the study was measuring their oral proficiency skills and so, highly engaged participants seeking to do their best in the task, those with high levels of agreeableness, provided as much detail as possible during the narrations which was matched with semantic gestures. During the exit interviews a number of participants reported trying to produce iconical gestures to ensure their utterances were clear. Their gesture production in informal contexts might not contain as many gestures. An additional variable might be culture, as speakers have been noted to gesture differently depending on their cultural background (Kita, 2009).

Participants also believed, based on teachers’ comments and online videos, that controlling their gestures enhanced their deliveries, inhibiting them if nervous and producing discursive ones, in particular, to show confidence. We suggest that flutters are micro-gestures that are actually speech-gestures which have been inhibited, and that younger and more self-aware individuals aim to minimize these and adaptors to seem more self-confident.

These explanations all point to external variables. In particular we would like to note that of task anxiety, the most studied emotion among language learners (Dörnyei and Ryan, 2015; Horwitz and Nassif, 2018). It is important to differentiate between trait anxiety, associated to neuroticism (an inclination to anxiety in general) and state anxiety, which is an emotional response to a specific set of circumstances, manifested as feelings of tension, apprehension, nervousness, and worry (Dörnyei, 2005). BFI results have also been noted to vary by context, as different situations might lead to state and situation-specific emotional responses. It is very likely that in our study participants, especially the younger ones, might have experienced significant levels of state Foreign Language Anxiety (FLA). Future studies could focus not on personality but on the levels of anxiety and enjoyment participants are experiencing during the data collection sessions. We strongly recommend that exit discussions are carried out to understand how participants felt and what their opinion on gestures might be.

## Data Availability

The datasets presented in this study can be found in online repositories. The names of the repository/repositories and accession number(s) can be found at: https://osf.io/yxurf/?view_only=2736e64987914bbfafb28cdbbd1c7fc6.

## Footnotes

^1^Explanation of the results in Table 2: For each dependent variable, a baseline model was first developed under the null hypothesis (H₀), which assumes that the independent variables (personality traits, gender, proficiency, and age) do not affect the dependent variable (type of gesture). This model serves as a reference point. The relationship between the dependent variable and independent variables is expressed in the form of a linear equation. This equation predicts the dependent variable based on the values of the independent variables. The goodness of fit of the model is evaluated by assessing how well the model explains the variance in the dependent variable. After fitting the model (H₁), the predicted results from the model are compared against the actual observed values of the dependent variable. This comparison helps to determine how well the model performs. Deviance measures how well the statistical model (H₁) fits the data compared to the baseline model (H₀). A lower deviance indicates a better fit of the model to the data. The Chi-square statistic is used to determine whether there is a significant difference between observed and expected frequencies in categorical data. A significant Chi-square value (with a corresponding *p*-value <0.05) indicates a significant association between variables. Akaike Information Criterion (AIC) helps to compare different models by considering both the goodness of fit and the number of parameters used. A lower AIC value indicates a better model when comparing multiple models. Bayesian Information Criterion (BIC) is similar to AIC but applies a heavier penalty for models with more parameters. A lower BIC value suggests a better fit. In this analysis, not all independent variables were found to have a significant effect on the dependent variable. Only those that were significant are reported in the right-hand side of Table 2. For the full set of results see https://osf.io/yxurf/?view_only=2736e64987914bbfafb28cdbbd1c7fc6.
